# Anoikis-related gene signature associates with the immune infiltration and predicts the prognosis of glioma patients

**DOI:** 10.1016/j.gendis.2024.101346

**Published:** 2024-06-04

**Authors:** Jianghua Lin, Junbao Wang, Junmiao Zhao, Xinyi Wu, Leiyu Hao, Xiao Tan, Lixue Yang, Lei-Lei Wu, Yuyang Xia, Xiaoling Zhang, Kaijun Zhao, Yu'e Liu

**Affiliations:** a Department of Neurosurgery, Shanghai East Hospital, School of Medicine, Tongji University, Shanghai 200120, China; b Yongkang First People's Hospital Affiliated to Hangzhou Medical College, Jinhua, Zhejiang 321300, China; c Tongji University Cancer Center, Shanghai Tenth People's Hospital of Tongji University, School of Medicine, Tongji University, Shanghai 200092, China; d Department of Biliary Tract Surgery Ⅱ, Eastern Hepatobiliary Surgery Hospital, Shanghai 201805, China; e Department of Thoracic Surgery, Shanghai Pulmonary Hospital, School of Medicine, Tongji University, Shanghai 200433, China; f School of Medicine, I.M. Sechenov First Moscow State Medical University, Moscow 119991, Russia; g National Joint Engineering Laboratory for Human Disease Animal Models, Key Laboratory of Organ Regeneration and Transplantation First Affiliated Hospital of Jilin University, Changchun, Jilin 130061, China

Glioma is the most aggressive and incurable brain tumor.^1^ Anoikis is programed apoptosis in which cells are detached from the cellular matrix and suspended to death.^2^ It plays a critical role in cancer progression. Cancer cells overcome anoikis via various methods to continue their progression and metastasis. But the role of anoikis in gliomas remains elusive. By comprehensively studying the glioma patient's data in public databases, we built an anoikis-related genes risk nomogram for glioma and validate independently. We found the high-risk groups were enriched in the anoikis and immune-related pathways. Further analysis of high-risk score groups revealed that they had higher immune infiltration, lower IDH-mutations, higher tumor mutation burdens and higher tumor immune dysfunction and exclusion scores, indicating that there might not be successful for the high-risk score glioma patients in immunotherapy. In conclusion, we built a risk modal by applying the analysis of anoikis-related genes and immune signature genes, and this risk modal can predict glioma patients' clinical outcomes successfully and accurately.

To decipher the role of anoikis in the prognosis of glioma, we first studied data from The Cancer Genome Atlas (TCGA). According to the expression of 320 anoikis-related genes (Table S1), we separated these patients into two groups by an unsupervised clustering approach (Fig. 1A; Fig. S1A–D). The 320 anoikis-related gene (ARG) expression significantly diverged across the two clusters. Patients in anoikis cluster 2 with higher anoikis-related gene expression had a worse prognosis than those in cluster 1 with lower ARG expression. And the overall survival (OS) revealed a substantial difference between these two clusters (Fig. 1B), suggesting that ARGs are crucial for glioma patients' prognosis.

**Figure 1:**
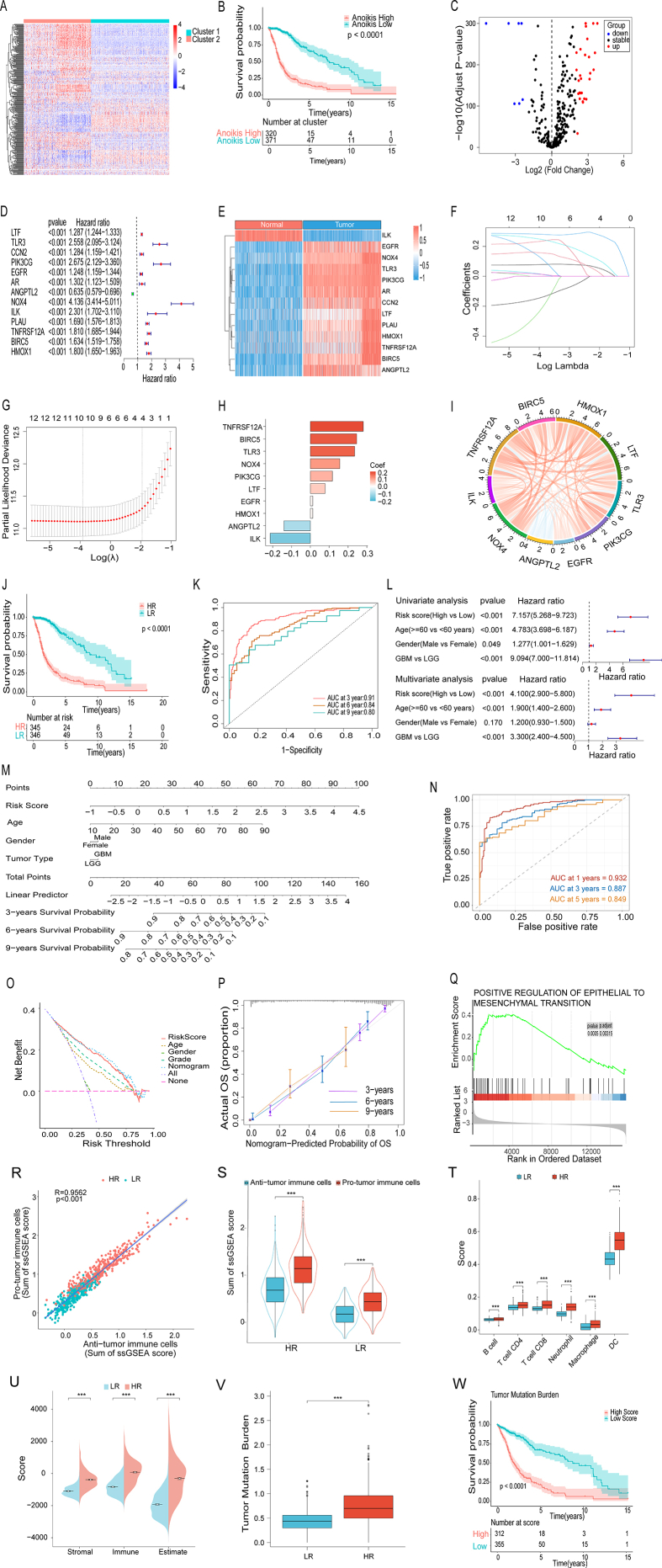
The construction and validation of an anoikis-related gene risk model in glioma. **(A)** Unsupervised clustering was used to construct heatmaps of anoikis-associated genes in two sample groups. **(B)** The Kaplan–Meier survival curve of two sample groups produced using unsupervised clustering. **(C)** TCGA and GTEx-based volcano map of glioma anoikis-associated differentially expressed genes. **(D)** A forest plot was created to characterize the prognosis of 13 immunological and anoikis-related genes throughout the glioma cohort. **(E)** Heat map of 13 immuno- and anoikis-related differential genes screened. **(F)** LASSO coefficient profiles of 13 genes. **(G)** In the LASSO model, a coefficient path graph is generated against the log (lambda) sequence. The optimal parameter (lambda) is selected as indicated by the first black dashed line. **(H)** In the LASSO model, coefficient of the 10 selected genes. **(I)** 10 genes' correlation plot of chords. **(J)** The curve of prognosis for glioma samples in TCGA databases. **(K)** In the TCGA data, the AUC of the time-dependent ROC curve validated the predictive performance of risk scores. **(L)** Univariate (top) and multivariate (bottom) COX regression analysis of the risk scores and a number of clinical variables in TCGA train set. **(M)** Creating a nomogram based on TCGA glioma patients' clinical data and risk ratings. **(N)** The AUC of the time-dependent ROC curve validated the prediction performance of the TCGA training set nomogram. **(O)** Decision curve analysis for TCGA data. **(P)** TCGA training set's calibration curves for 3-year, 6-year, and 9-year survival nomograms. A better guess is indicated by a tighter match to the diagonal gray line. **(Q)** GSEA indicates that the pathway of positive regulation of epithelial to mesenchymal transition was enriched in the High-Risk group. **(R)** Correlation between anti-tumor immune cells (ActCD4, ActCD8, TcmCD4, TcmCD8, TemCD4, TemCD8, Th1, Th17, ActDC, CD56briNK, NK, NKT) and pro-tumor immune (Treg, Th2, CD56dimNK, imDC, TAM, MDSC, Neutrophil, and pDC) cells. The darkened region reflects a confidence interval with a 95% level of confidence. **(S)** Variations in the abundance of anti-tumor and pro-tumor immune cells between the LR and HR groups. **(T)** The TIMER algorithm-obtained ratings of immune cells were compared in HR and LR. **(U)** Comparison of HR and LR groups' stromal scores, immune scores, and ESTIMATE scores. **(V)** The connection between risk scores and TMB. **(W)** Prognosis of TMB grouping.

To check the accurate ARGs which determine the glioma growth, we then investigated gene expression differences between TCGA and GTEx samples, and found 37 differentiated genes (DEGs), among which, 30 are up-regulated and 7 are down-regulated (Fig. 1C). Since the anoikis interacted with the immune system to promote cancer metastasis, we thus compared these 37 DEGs with 2483 genes with “immunological signatures” and found 15 genes are in the intersection (Fig. S1E), among which, 13 genes related with anoikis were substantially associated with the OS of glioma patients (Fig. 1D). Most of them are highly expressed in the tumor group (Fig. 1E).

We then developed a prognostic model by LASSO Cox regression analysis. After the evaluation of the best possible value of λ, a signature of 10 genes was established (Fig. 1F–H). Most of them were shown to be positively related with each other (Fig. 1I). Furthermore, the influence of gene expression with greater absolution value of risk coefficients on the prognosis of glioma patients was investigated, and the findings revealed that TNFRSF12A, NBIRC5, and TLR3 were prognostic risk factors, whereas ANGPTL2 was a protective factor for prognosis in glioma patients (Fig. S2A–E). Then, a risk score was calculated using the following formula: risk score = (0.08∗LTF exp.) + (0.24∗LTR3 exp.) + (0.12∗PIK3CG exp.) + (0.01∗EGFR exp.) + (−0.14∗ANGPTL2 exp.) + (0.16∗NOX4 exp.) + (−0.21∗ILK exp.) + (0.28∗TNFRSF12A exp.) +(0.25∗BIRC5 exp.) +(0.01∗HMOX1 exp.). Meanwhile, following the determination of the median risk score, patients were sorted into HR (high-risk) group and LR (low-risk) group. HR patients in the TCGA cohort had significantly higher mortality rates than LR individuals (Fig. S2F). All of the OS rate of HR patients was much lower than that of LR patients across all other categories (Fig. 1J). Principal component analysis (PCA) revealed that LR and HR groups were efficiently separated (Fig. S2G). The Area Under Curve (AUC) indicates the high sensitivity and specificity of this risk score system (Fig. 1K). We then verified the result via CGGA data (Fig. S2H–K). The findings demonstrated a strong correlation between risk score, age, and tumor grade with the prognosis of glioma patients in both the TCGA and CGGA cohorts (Fig. 1L; Fig. S3A). To demonstrate the clinical applicability of risk models, we constructed clinical nomograms of TCGA (Fig. 1M) and CGGA cohort (Fig. S3B), which can predict glioma survival probability based on risk scoring models. The nomogram's time-dependent ROC displays AUC (Fig. 1N; Fig. S3C). The Decision curve analysis (DCA) findings demonstrated that our risk model gave the largest net profit (Fig. 1O; Fig. S3D). The calibration plot revealed that the training and validation sets had a high level of concordance between the OS prediction and observation possibilities (Fig. 1P; Fig. S3E), indicating the nomogram is quite precise. In the TCGA training set, the nomogram had the highest predictive value compared to the risk score and clinical characteristics (Fig. S3F–H). The CGGA validation set nomogram also had the greatest projected value (Fig. S3I–K).

We then checked the relation between risk models and clinical indicators, all samples were divided into subgroups depending on patient clinical features. High-risk (HR) patients' survival curves were consistently worse than those of low-risk (LR) patients across all categories (Fig. S4A–F). And high-risk individuals were older and more malignant (Fig. S4G–H). Tumor in high-grade individuals is more likely to have a higher recurrence rate (Fig. S4I). Moreover, the risk score was substantially negatively connected with IDH mutations (Fig. S4J), whereas lp/19q codeletion was mostly detected in low-risk individuals (Fig. S4K). Therefore, we concluded that our risk model is also capable of differentiating and predicting the prognosis of certain subgroups.

To further assess the functioning of the risk model, we did a differential analysis (Fig. S5A) on the two groups of patients (HR group and LR group) and performed KEGG and GO studies on DEGs. DEGs between the HR and LR groups were enriched for many functions associated to anoikis and immunity (Fig. S5B–E). GSEA analysis indicated that the HR group was enriched in positive regulatory pathway of epithelial interstitial transformation, which is closely associated with anoikis (Fig. 1Q). Surprisingly, the HR group was abnormally active in immune-related activities (Fig. S5F–H), while immunosuppressive pathways (Fig. S5I–K), were also enriched in the HR group.

We then conducted single sample gene set enrichment analysis (ssGSEA) and found the HR group exhibited higher degree of immune cell infiltration (Fig. S6A). We next divided these immune cells into pro- and anti-tumor ones and found there is a substantial positive association between the quantity of pro- and anti-tumor immune cells in the glioma environment (Fig. 1R). Moreover, the quantity of tumor-promoting immune cells was larger in the HR and LR groups than that of anti-tumor immune cells (Fig. 1S). We also evaluated the immune cell infiltration of glioma samples using the TIMER^3^ (Tumor Immune Estimation Resource) and found that patients in the HR group had higher immune cell scores than those in the LR group (Fig. 1T; Fig. S6B). In addition, we used the ESTIMATE^4^ (Estimation of Stromal and Immune Cells in Malignant Tumors using Expression data) method to calculate the immune infiltration scores and found ESTIMATION score of patients with high risk ratings were higher (Fig. 1U). We then tested Tumor Mutation Burden (TMB) and found that high-risk individuals had greater TMB, which was consistent with the findings of the earlier studies (Fig. 1V).^5^ An examination of patients' survival rates revealed that those with reduced TMB had a more favorable prognosis (Fig. 1W).

In conclusion, we designed a risk modal that can predict the glioma patient's prognosis and clinical outcomes successfully by applying the function of anoikis-related genes in glioma.

## Author contributions

Y.L. and K.Z. designed the project and wrote partial the manuscript. J.L did data analysis and wrote partial of the article. J.W analyzed the data partially. J.Z, X. W, L.H and X.T sourced the literatures, L.Y, X.Z and X.Z polished the language. All authors contributed to the article, read and approved the article.

## Availability of data and material

Clinical data and RNA-seq data were gathered from the TCGA cohort and the GTEx cohort in UCSC Xena (https://xenabrowser.net/), respectively. The TCGAbiolinks software package supplied with R software is used to gather mutation spectrum data from the TCGA database (https://portal.gdc.cancer.gov/). The validation dataset was retrieved from the CGGA database 693 queue (http://www.cgga.org.cn). The GeneCards website (https://www.genecards.org) was used to obtain 338 anoikis genes with a correlation value of >1.0 as the cutoff. Genes associated to immunity were downloaded from the ImmPort website (https://www.immport.org/).

## Conflict of interests

The authors declare that they have no competing interests.

## Funding

This study was funded Pudong New Area Health Commission (PW2022A-28) and Neuroscience Innovation and Development Research Project (YXJL-2022-00351-0183).

## Appendix A Supplementary data

Multimedia component 1 Multimedia component 1

Multimedia component 2 Unsupervised clustering of anoikis-related genes in glioma. (A) Plot of Item-Consensus at k=2 and k=3, with k=2 being the best. (B) The cumulative distribution function ranges from K=2 to 10, with the optimal k-value being the smoothest upward slope. (C) A consensus matrix with K equal to 2 demonstrates that samples are solidly grouped into two groups. (D) Consensus matrix with K equal to 3. (E) Venn diagram used to screen differentially expressed genes associated with immunity and anoikis. Multimedia component 2

Multimedia component 3 Verification of risk models. Prognostic curves for genes associated with poor prognosis, TNFRSF12A (A), NBIRC5 (B), and TLR3 (C), alongside survival curves for genes indicative of favorable prognosis, ANGPTL2 (D) and ILK (E). (F) Survival rates (top) and risk scores (bottom) for glioma patients in the TCGA database. (G) PCA plot of the TCGA data. (H) Survival rates (top) and risk scores (bottom) for glioma patients in the CGGA database. (I) Curve of prognosis for glioma samples in CGGA databases. (J) PCA plot of the CGGA data. (K) In the CGGA data, the AUC of the time-dependent ROC curve validated the predictive performance of risk scores. Multimedia component 3

Multimedia component 4 Development of a nomogram for survival prediction. (A) On the risk score and other clinical factors of the CGGA validation set, univariate (top) and multivariate (bottom) COX regression analyses were conducted. (B) Nomogram of clinical data and risk score for patients with CGGA glioma. (C) The AUC of the time-dependent ROC curve validated the prediction performance of the CGGA validation set nomogram. (D) Decision curve analysis for CGGA data. (E) CGGA validation set’s calibration curves for 3-year, 6-year, and 9-year survival nomograms. (F-H) ROC curve based on the exponent of a nomogram in TCGA cohort. (I-K) ROC curve based on the exponent of a nomogram in CGGA cohort. Multimedia component 4

Multimedia component 5 Risk model correlation with clinical features. (A) Survival curve for glioma patients in TCGA database over 60, classified by risk scores (HR: high-risk, LR: low-risk), (B) Survival curve for glioma patients under 60 years old with high- and low- risk score, data from TCGA. (C) Survival curve for female glioma patients with high- and low- risk score, data from TCGA. (D) Survival curve for male glioma patients with high- and low- risk score. (E) First-onset patients' survival curves in the CGGA validation set. (F) Relapsed patients' prognostic curves in the CGGA validation set. Risk score correlations with age (G),and tumor type (H) in TCGA train set. Risk score correlations with recurrent conditions (I), IDH mutation status (J) and 1p/19q codeletion (K) in CGGA validation set. Multimedia component 5

Multimedia component 6 Functional analysis enhanced items. (A) The volcano graphic depicted the differences in genes between patients in the high and low risk categories. (B-C) Exhibition of DEGs' GO Enrichment Pathway. Both figures share the same legend. (D-E) KEGG analysis yielded the following major terms. Both figures share the same legend. (F-K) GSEA findings for differential gene set enrichment in high-risk patients. FigureS6 The connection between a risk score and immunological function. (A) ssGSEA scores for immune cells. (B) Comparison of immune cell ratings produced by the EPIC algorithm in HR and LR. Multimedia component 6

Multimedia component 7 The connection between a risk score and immunological function. ssGSEA scores for immune cells. (B) Comparison of immune cell ratings produced by the EPIC algorithm in HR and LR. Multimedia component 7
