# Supplementary material for: Anoikis-related gene signature associates with the immune infiltration and predicts the prognosis of glioma patients
Source: Genes Dis. 2024 Jun 4;12(2):101346. doi: 10.1016/j.gendis.2024.101346 (PMC11625316; doi:10.1016/j.gendis.2024.101346)
Supplement: Multimedia component 1 [file mmc1.docx]

**Materials and methods**

**Data Collection**

### Clinical data and RNA-seq data were gathered from the TCGA cohort and the GTEx cohort in UCSC Xena (https://xenabrowser.net/), respectively. The TCGAbiolinks software package supplied with R software is used to gather mutation spectrum data from the TCGA database (https://portal.gdc.cancer.gov/). The validation dataset was retrieved from the CGGA database 693 queue (http://www.cgga.org.cn). Data from WHO grade I glioma patients were not gathered since these tumors are benign. The GeneCards website (https://www.genecards.org) was used to obtain 338 anoikis genes with a relevance scores of >1.0 as the cutoff^17^. Genes associated to immunity were downloaded from the ImmPort website (https://www.immport.org/).

**Unsupervised grouping of expression genes related with anoikis**

R-package “ConsensusClusterPlus” was used to perform unsupervised consensus clustering of anoikis-related genes using K-means^18^. sampled 80% of the data each time, and identified k-values that grouped the samples appropriately. The R package "survival" was used to conduct the survival analysis in this article.

**Screening for anoikis and immune-related DEGs**

Using the “limma” package^19^, differential analysis was done between normal samples from GTEx and glioma samples from TCGA. The adjusted *p* value < 0.05 and |Log2 (Fold Change)| > 2 were utilized to search for anoikis-related differential genes, and the intersection of immune-related genes and anoikis-related differential genes was obtained. The volcano map was generated using the "ggplot2" R tool, while the heat map was generated using the "pheatmap" R program.

**Model construction for prognostic risk scoring**

In the TCGA dataset, the correlation between differential gene expression and overall survival (OS) was calculated using univariate Cox proportional regression analysis. Next, relevant genes were included in the R-pack "glmnet" LASSO analysis with *p* <0.05^20^. The minimal penalty (λ) was chosen using tenfold cross-validation, and the anoikis associated risk model was built: risk score = (A gene expression * coefficient + B gene expression * coefficient + C gene expression * coefficient .etc). Set a cutoff point for the median risk score to distinguish between high-risk and low-risk patient groups. PCA is carried out using the "prcomp" function of the R package "factoextra", and a time-dependent ROC curve analysis was performed using the "survival ROC" R tool to assess the predictive ability of the risk model. For correlation analysis and visualization, the R package “circlize” and “ggcorrplot” were employed.

**Risk score correlation with clinical parameters**

We used R package “survival” to run univariate Cox regression analysis to evaluate risk scores as independent prognostic variables. Built the nomgram of risk scores and clinicopathological features using the R package "rms". The "survival ROC" R package is used to study and create time-dependent ROC curves to evaluate the accuracy of risk model forecasts. Using the R package "ggDCA", decision curve analysis (DCA) was performed on a multivariate Cox regression model.

**Functional enrichment analysis**

Using the "clusterprofiler" R package, a full functional enrichment study was undertaken, including analyses of GO enrichment, KEGG enrichment, and GSEA enrichment. The GO and KEGG findings were visualized using the R tool "circlize". The MSigDB website (http://www.gsea-msigdb.org/) provided with annotation data for GSEA analysis.

**Immunoassay for risk models**

The degree of immune cell infiltration in glioma samples was assessed using ssGSEA analysis with R-pack "GSVA."^21^ In immune infiltration analysis, TIMER (Tumor Immune Estimation Resource), EPIC (Estimating the Percentage of Immune and Cancer Cells), and ESTIMATE (Estimation of Stromal and Immune Cells in Malignant Tumors using Expression data) were utilized. All of the algorithms are from the “IOBR” package^22^. R package “maftools” was used to analyze and show somatic variation data from GBM and LGG patients. TMB (mutations per million bases) were then determined for each patient. TIDE is a computational tool for predicting the clinical response of ICB and assessing the possibility of tumor immune escape^23^. TIDE score and ICB responses in LGG and GBM patients were examined using the TIDE website (http://tide.dfci.harvard.edu/) after inputting standardized expression profile data. The Xiantao Academic website (https://www.xiantao.love/) created all grouping comparison charts and pod diagrams. The Wilcoxon rank-sum test was performed to compare patients in the high-risk group with those in the low-risk group, and the P value < 0.05, which indicated that there was a statistically significant difference between the two groups.
