# Supplementary figures and images for: Anoikis-related gene signature associates with the immune infiltration and predicts the prognosis of glioma patients
Source: Genes Dis. 2024 Jun 4;12(2):101346. doi: 10.1016/j.gendis.2024.101346 (PMC11625316; doi:10.1016/j.gendis.2024.101346)

Supplementary Figure 1

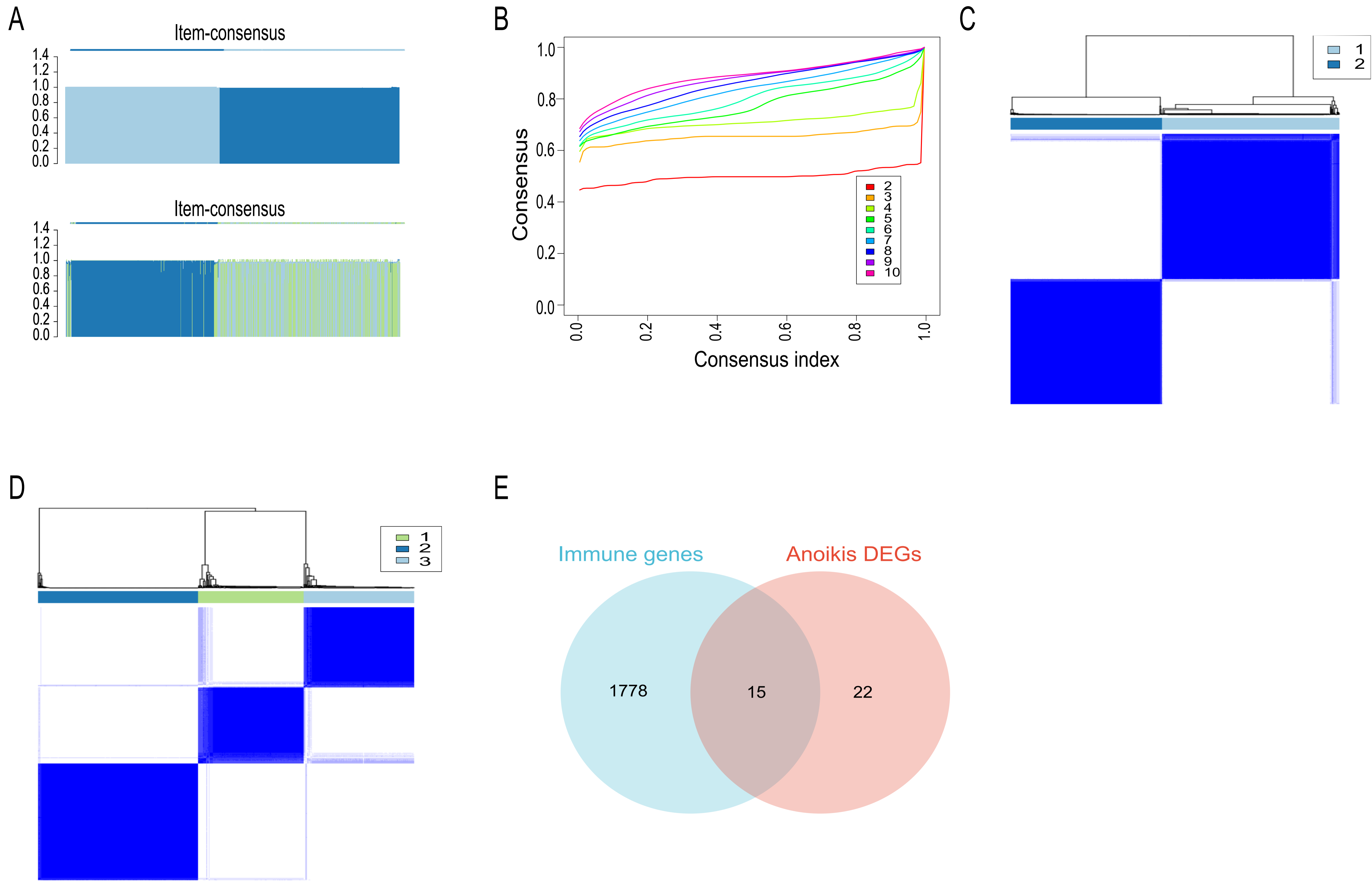

Supplement: Multimedia component 2 — Unsupervised clustering of anoikis-related genes in glioma. (A) Plot of Item-Consensus at k=2 and k=3, with k=2 being the best. (B) The cumulative distribution function ranges from K=2 to 10, with the optimal k-value being the smoothest upward slope. (C) A consensus matrix with K equal to 2 demonstrates that samples are solidly grouped into two groups. (D) Consensus matrix with K equal to 3. (E) Venn diagram used to screen differentially expressed genes associated with immunity and anoikis. [file mmc2.pdf]

Supplementary Figure 2

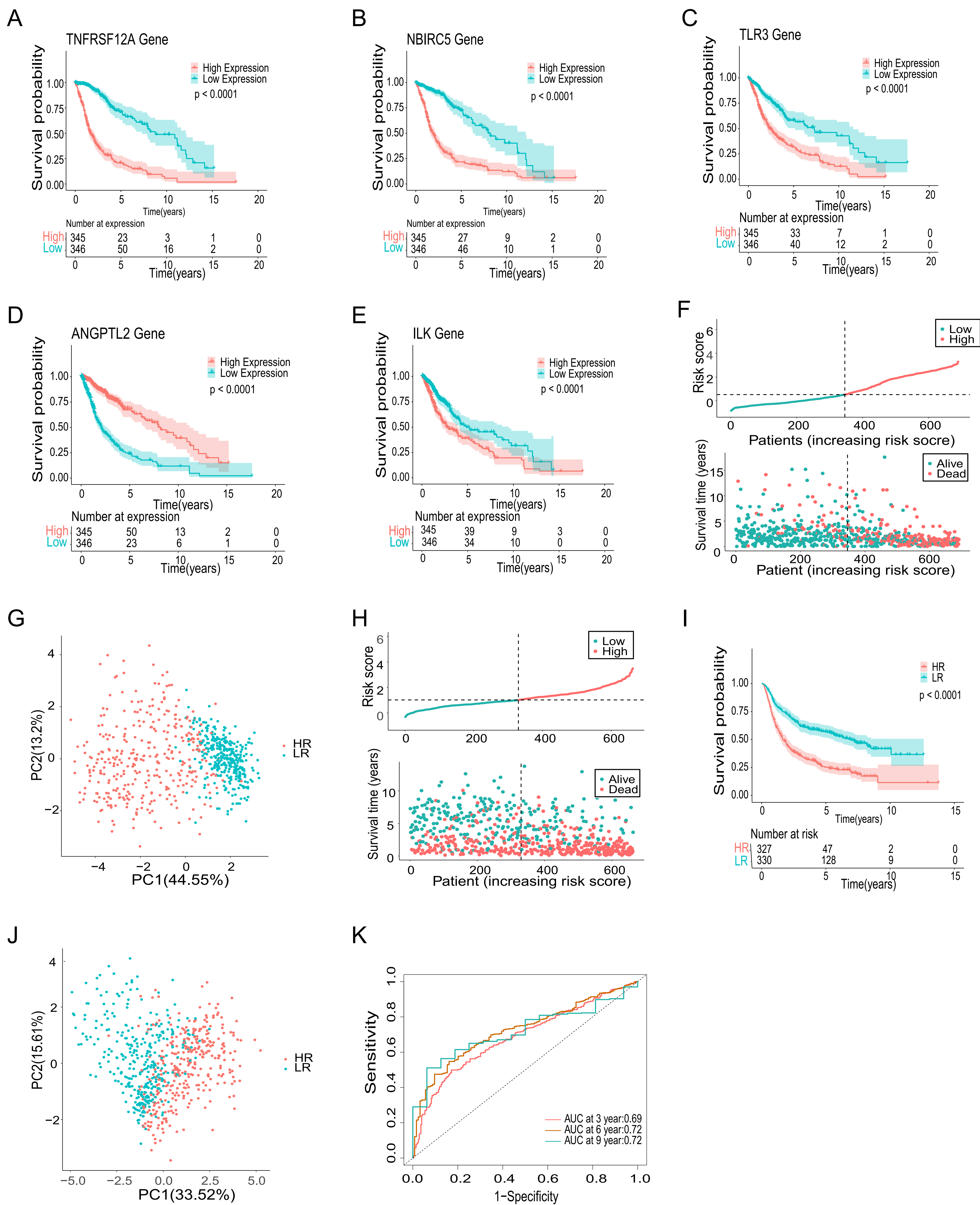

Supplement: Multimedia component 3 — Verification of risk models. Prognostic curves for genes associated with poor prognosis, TNFRSF12A (A), NBIRC5 (B), and TLR3 (C), alongside survival curves for genes indicative of favorable prognosis, ANGPTL2 (D) and ILK (E). (F) Survival rates (top) and risk scores (bottom) for glioma patients in the TCGA database. (G) PCA plot of the TCGA data. (H) Survival rates (top) and risk scores (bottom) for glioma patients in the CGGA database. (I) Curve of prognosis for glioma samples in CGGA databases. (J) PCA plot of the CGGA data. (K) In the CGGA data, the AUC of the time-dependent ROC curve validated the predictive performance of risk scores. [file mmc3.pdf]

Supplementary Figure 3

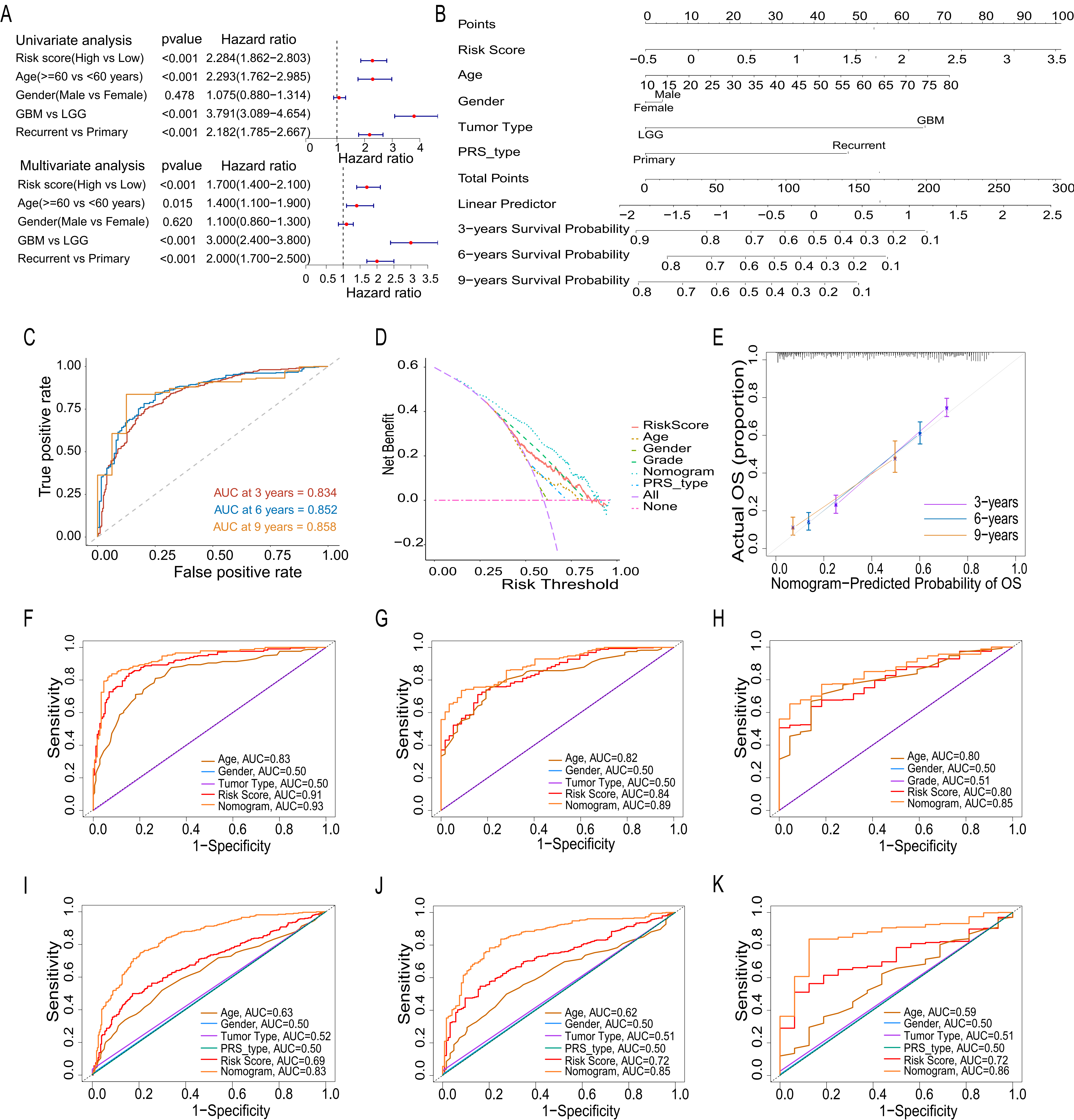

Supplement: Multimedia component 4 — Development of a nomogram for survival prediction. (A) On the risk score and other clinical factors of the CGGA validation set, univariate (top) and multivariate (bottom) COX regression analyses were conducted. (B) Nomogram of clinical data and risk score for patients with CGGA glioma. (C) The AUC of the time-dependent ROC curve validated the prediction performance of the CGGA validation set nomogram. (D) Decision curve analysis for CGGA data. (E) CGGA validation set’s calibration curves for 3-year, 6-year, and 9-year survival nomograms. (F-H) ROC curve based on the exponent of a nomogram in TCGA cohort. (I-K) ROC curve based on the exponent of a nomogram in CGGA cohort. [file mmc4.pdf]

Supplementary Figure 4

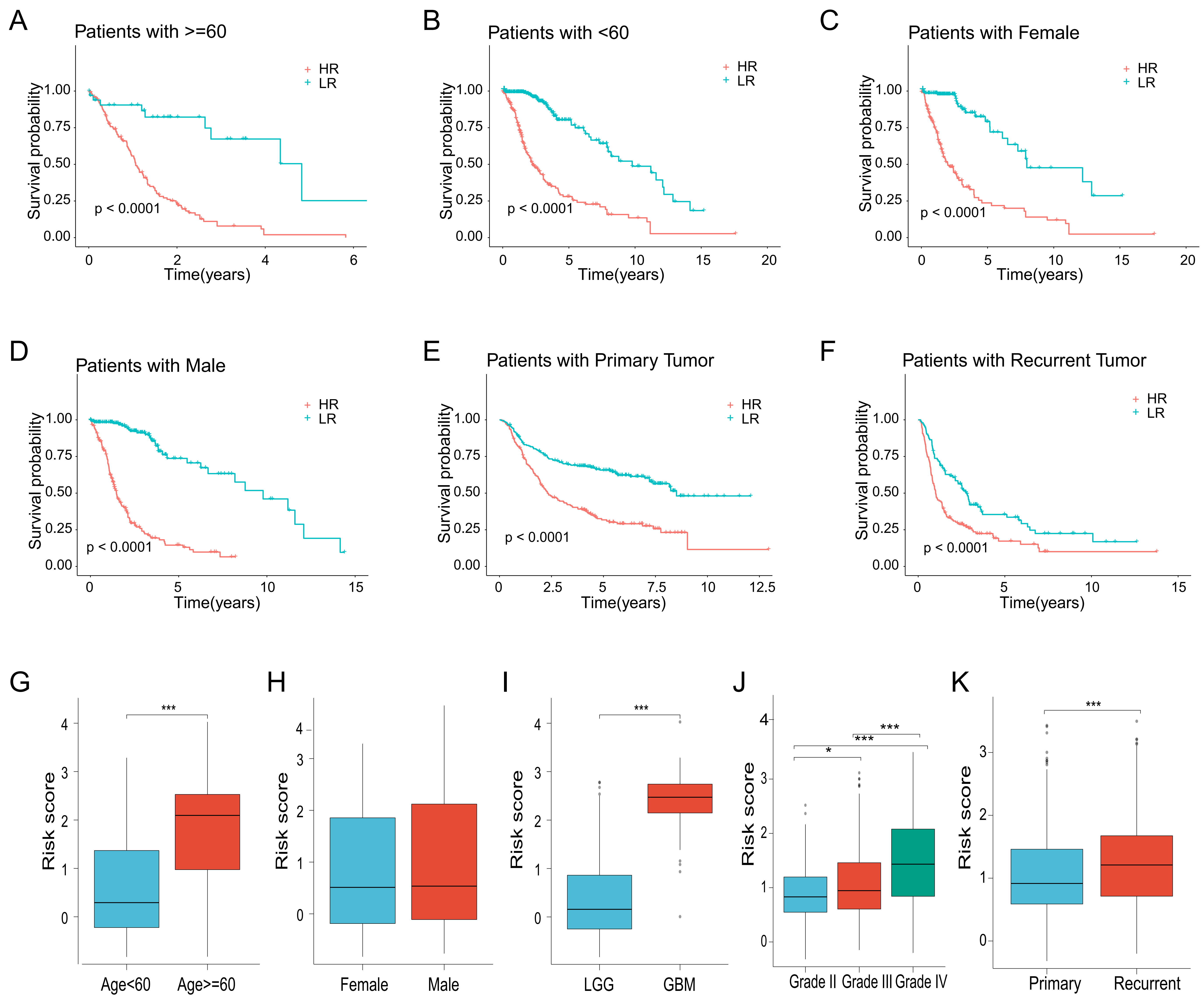

Supplement: Multimedia component 5 — Risk model correlation with clinical features. (A) Survival curve for glioma patients in TCGA database over 60, classified by risk scores (HR: high-risk, LR: low-risk), (B) Survival curve for glioma patients under 60 years old with high- and low- risk score, data from TCGA. (C) Survival curve for female glioma patients with high- and low- risk score, data from TCGA. (D) Survival curve for male glioma patients with high- and low- risk score. (E) First-onset patients' survival curves in the CGGA validation set. (F) Relapsed patients' prognostic curves in the CGGA validation set. Risk score correlations with age (G),and tumor type (H) in TCGA train set. Risk score correlations with recurrent conditions (I), IDH mutation status (J) and 1p/19q codeletion (K) in CGGA validation set. [file mmc5.pdf]

Supplementary Figure 5

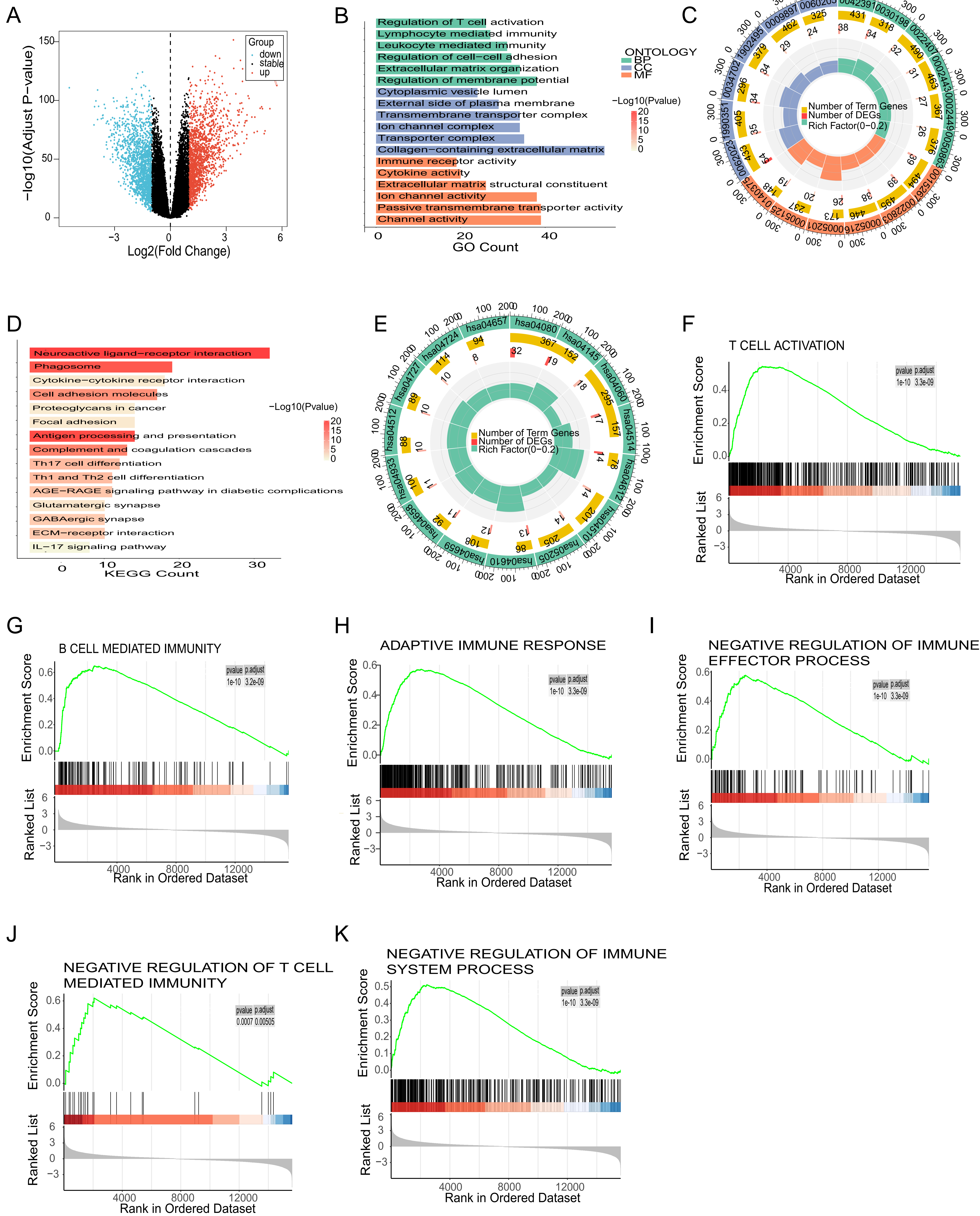

Supplement: Multimedia component 6 — Functional analysis enhanced items. (A) The volcano graphic depicted the differences in genes between patients in the high and low risk categories. (B-C) Exhibition of DEGs' GO Enrichment Pathway. Both figures share the same legend. (D-E) KEGG analysis yielded the following major terms. Both figures share the same legend. (F-K) GSEA findings for differential gene set enrichment in high-risk patients. FigureS6 The connection between a risk score and immunological function. (A) ssGSEA scores for immune cells. (B) Comparison of immune cell ratings produced by the EPIC algorithm in HR and LR. [file mmc6.pdf]

Supplementary Figure 6

A

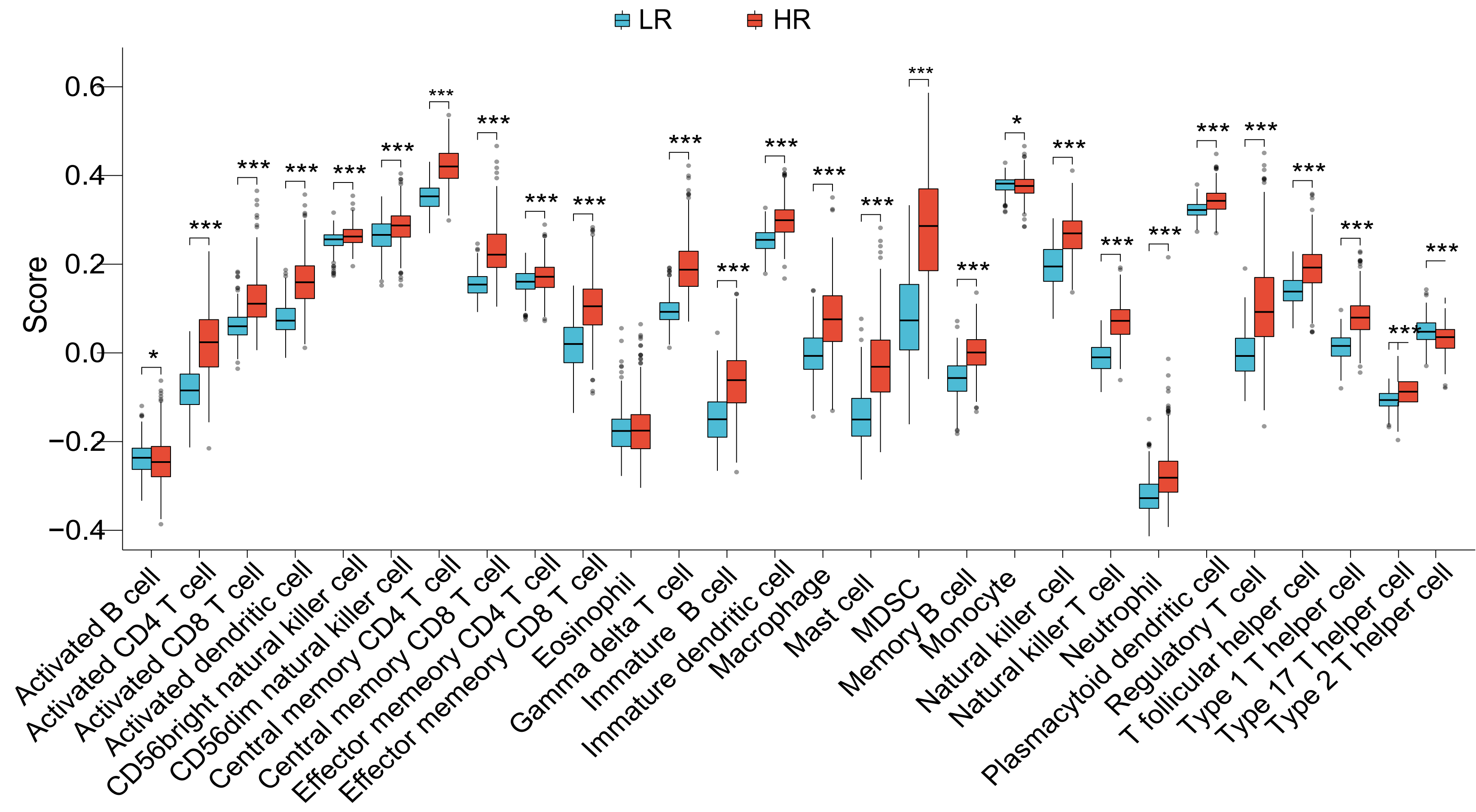

B

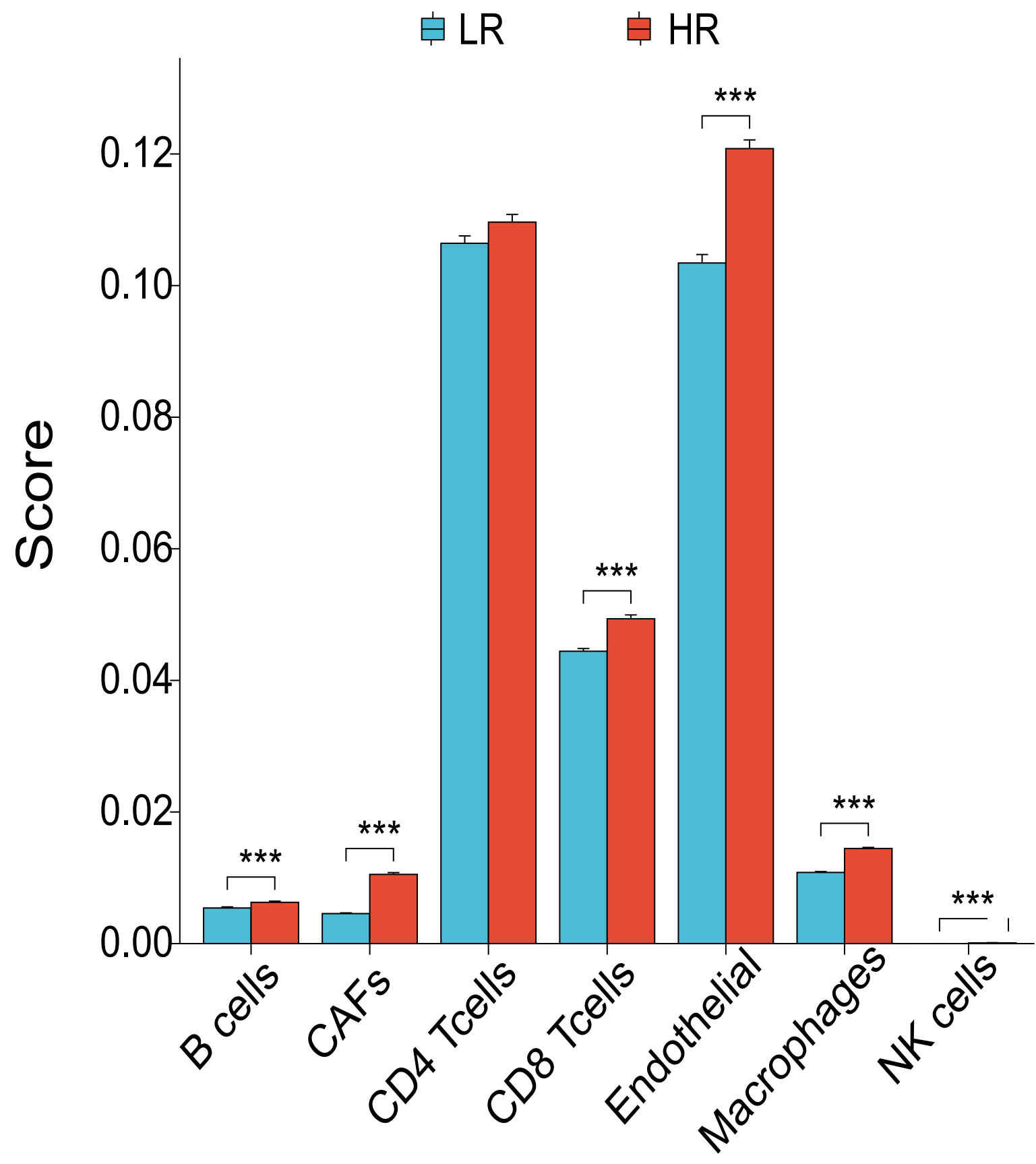

Supplement: Multimedia component 7 — The connection between a risk score and immunological function. ssGSEA scores for immune cells. (B) Comparison of immune cell ratings produced by the EPIC algorithm in HR and LR. [file mmc7.pdf]
